# Modeling Coding Intensity of Procedures in a U.S. Population-Based Hip/Knee Arthroplasty Inpatient Cohort Adjusting for Patient- and Facility-Level Characteristics

**DOI:** 10.3390/healthcare10081368

**Published:** 2022-07-23

**Authors:** Nancy G. Rios, Paige E. Oldiges, Marcela S. Lizano, Danielle S. Doucet Wadford, David L. Quick, John Martin, Michael Korvink, Laura H. Gunn

**Affiliations:** 1 School of Data Science, University of North Carolina at Charlotte, Charlotte, NC 28223, USA; nriosais@uncc.edu (N.G.R.); poldiges@uncc.edu (P.E.O.); mlizano@uncc.edu (M.S.L.); ddoucet@uncc.edu (D.S.D.W.); 2 Department of Public Health Sciences, University of North Carolina at Charlotte, Charlotte, NC 28223, USA; dquick1@uncc.edu; 3 ITS Data Science, Premier, Inc., Charlotte, NC 28277, USA; john_martin@premierinc.com (J.M.); michael_korvink@premierinc.com (M.K.); 4 School of Public Health, Faculty of Medicine, Imperial College London, London W6 8RP, UK

**Keywords:** coding intensity, MS-DRG, ICD-10, procedures, hip-knee arthroplasty, risk adjustment

## Abstract

Variations in procedure coding intensity, defined as excess coding of procedures versus industry (instead of clinical) standards, can result in differentials in quality of care for patients and have additional implications for facilities and payors. The literature regarding coding intensity of procedures is limited, with a need for risk-adjusted methods that help identify over- and under-coding using commonly available data, such as administrative claims. Risk-adjusted metrics are needed for quality control and enhancement. We propose a two-step approach to risk adjustment, using a zero-inflated Poisson model, applied to a hip-knee arthroplasty cohort discharged during 2019 (*n* = 313,477) for patient-level risk adjustment, and a potential additional layer for adjustment based on facility-level characteristics, when desired. A 21.41% reduction in root-mean-square error was achieved upon risk adjustment for patient-level factors alone. Furthermore, we identified facilities that over- and under-code versus industry coding expectations, adjusting for both patient-level and facility-level factors. Excess coding intensity was found to vary across multiple levels: (1) geographically across U.S. Census regional divisions; (2) temporally with marked seasonal components; (3) by facility, with some facilities largely departing from industry standards, even after adjusting for both patient- and facility-level characteristics. Our proposed method is simple to implement, generalizable, it can be used across cohorts with different sets of information available, and it is not limited by the accessibility and sparsity of electronic health records. By identifying potential over- and under-coding of procedures, quality control personnel can explore and assess internal needs for enhancements in their health delivery services and monitor subsequent quality improvements.

## 1. Introduction

The International Classification of Diseases (ICD) has a wide range of applications worldwide and provides essential information on the scope, causes, and consequences of human disease and death [1]. Payment systems, service planning, quality and safety management, and health services research benefit from this data [1]. ICD-9 and ICD-10 codes have been used worldwide and contributed to over 20,000 scientific publications [2]. Healthcare authorities and hospitals rely on standardized coding of medical diagnoses and procedures to perform epidemiological studies and calculate medical reimbursement costs [3,4]. This coding process, referred to as clinical abstraction of diagnoses and procedures during inpatient stays, is primarily conducted by humans and therefore subject to coding intensity variation [4].

The Centers for Medicare and Medicaid Services (CMS) have increasingly focused on coding intensity of diagnoses due to the substantial financial incentives for plans to record as many illnesses as they can identify [5]. While hospitals are evaluated using risk-adjusted quality measures [6,7,8], there is also a need to include risk-adjustment models for assessing coding intensity, which may be affected not only by patient characteristics, but also by processes and policies defined by providers. CMS uses diagnoses and patient demographics in their quality performance metrics, though with no clear focus on proper coding for procedures, which remain largely unexplored [9].

The large number of available codes increases the difficulty for clinical coders to maintain coding accuracy [10]. Variations in coding intensity, and miscoding in particular, can have serious implications for both payments and quality of care. Miscoding occurs due to a multitude of reasons, such as inaccuracy in coding assignments and omission of postoperative complications [11]. Failure to document all procedural work performed results in a significantly reduced hospital reimbursement, also known as under-coding [11]. For example, an audit of ICD-10 codes related to hepatopancreatic-biliary surgeries over a span of three months identified that over a third of total procedures were not coded [12]. Contrariwise, professionals coding above the services performed or substituting codes for procedures not covered by a payor for covered ones is known as upcoding or over-coding [13]. Over-coding leads to billions in additional public spending while significantly misrepresenting organizational behavior and patient needs [14]. While the literature provides instances of under-coding [11,12,13], the lack of findings pertaining to over-coding is somewhat expected due to the negative connotations associated, such as medical billing fraud, which is subject to severe penalties, or unnecessary/uncommon procedures for the patient characteristics [14,15,16].

The current literature does not sufficiently focus on assessing the appropriate number of additional procedures or diagnoses within a patient visit. Instead, the focus has been on developing risk-adjustment models concentrating on health outcomes [6,7,8,17,18]. These models do not address coding intensity. Simple approaches were used to predict cost variation in spine surgical procedures within the health communities, an advantage of which was identifying providers’ coding intensity and providing hospital savings [19]. However, the resulting model was disease specific, and a more generic and extrapolatable approach is needed to detect coding intensity differences against industry standards across diseases and procedures.

The approach presented in this manuscript assesses procedure coding intensity demonstrated through a cohort of total hip arthroplasty (THA) and total knee arthroplasty (TKA) procedures in combination with multiple patient- and facility-level characteristics commonly available through administrative claims data. Rates of THA and TKA procedures were predicted to increase by 174% and 673%, respectively, from 2005 to 2030, due to aging populations [20]. Information extracted from administrative claims data, which is all that may be available across some facilities and organizations, becomes increasingly important to inform quality control, resource utilization, and cost metrics [21]. By identifying several types of surgeries, ICD-10 codes provide high levels of granularity to hip and knee arthroplasty (and other) procedures [22].

Appropriate adjustment for patient and facility characteristics has the potential to enhance patient outcomes and underlying healthcare processes which may depart from industry standards. Our proposed approach fills a substantial gap in the literature by providing generalizable metrics for assessing coding intensity of procedures against industry standards of practice. These metrics can flag patient visits and facilities experiencing potential under- and over-coding, thus impacting patient safety outcomes and hospital quality performance. We demonstrate the approach using acute inpatient administrative claims data.

## 2. Methods

### 2.1. Data

Data from the Premier Healthcare Database (PHD) was used for this analysis and was provided by Premier, Inc. The PHD is a major hospital-based, service-level, private all-payor database in the U.S. that contains information on inpatient discharges [23]. Administrative, healthcare utilization, and financial data from patient visits are submitted by hospitals and healthcare systems. The information in the dataset used for this study includes 315,867 observations from acute inpatient hospital stays with Medicare Severity Diagnosis Related Group (MS-DRG) codes 461, 462, 466, 467, 468, 469, and 470, corresponding to hip or knee replacement, revision, or reattachment [24] (See Supplementary Table S1 for descriptions of THA/TKA MS-DRGs). The data extracted consists of the following: (1) counts of additional procedures reported, representing the outcome variable; (2) patient-level characteristics (age, sex, race, length of stay, primary payor, point of origin, ICD-10 principal diagnosis code, MS-DRG code, discharge status, and Agency for Healthcare Research and Quality (AHRQ) overall tract summary); (3) facility-level characteristics (masked facility ID, teaching status, urban or rural status, ownership status, academic status, bed count, Census region, and the hospital case mix index (CMI)); (4) admission month of the year corresponding to the patient visit. Many of these variables were provided in ranges for confidentiality purposes.

### 2.2. Statistical Analysis

Missing data was found in 0.76% of patient visits, and thus, we performed a complete case analysis comprising *n* = 313,477 complete observations. Descriptive statistics were calculated for all 19 variables contained in the dataset. Categories with observed frequencies below 0.1% were grouped together. Due to the large number of zero counts for the outcome variable, and potential for even larger cases in other cohorts, a zero-inflated Poisson model was proposed.

Controlling for patient-level characteristics, Equation (1) represents the zero-inflated Poisson model for the counts of additional ICD-10 procedure codes:

log(π[i]/(1 − π[i])) = α_1_+**γ** × PL[i], where π[i] = Prob(Y[i] = 0|PL[i]) Y[i]~Poi(λ[i]) with probability 1 − π[i], where log(λ[i]) = α_2_ + **β** × PL[i], (1)

where π[i] represents the risk-adjusted (inflated) probability of zero observed additional procedure counts for patient i (Y[i] = 0), which is modeled through a logit link as an overall mean α_1_ and a set of coefficients **γ** associated with each of the patient-level covariates (PL) for individual i. Conversely, with probability 1 − π[i], the observed counts of additional procedures Y[i] for individual i are assumed to follow a Poisson distribution, represented as Poi(), with individual-specific, risk-adjusted mean rate λ[i]. The log-mean rate of procedures is linked to an overall mean coefficient α_2_ and the aforementioned individual-specific, patient-level characteristics (PL[i]) with corresponding coefficients **β**. Both components of the zero-inflated model are assumed to be potentially associated with the same set of covariates.

For each patient i, Equation (2) estimates the excess coding intensity (ECI) as the observed counts (Y) of additional procedures for that patient visit minus the expected value (E) of the additional procedure counts given the patient-level characteristics derived using Equation (1):

ECI[i] = Y[i] − E(Y[i]|PL[i]).(2)

This estimated excess coding intensity for patient i, defined in Equation (2), is assumed to be normally distributed (see Equation (3)), expressed as N() with facility-level characteristics of the facility attended by patient i (FL[i]), a shared vector of coefficients **θ,** and a joint error term σ^2^ representing the variability in excess coding intensity not explained by facility-level characteristics (i.e., common inter-facility unexplained variability). Other distributional approaches are possible.

ECI[i]~N(**θ** × FL[i], σ^2^) (3)

Additionally, for each patient i, an adjusted excess coding intensity (AECI) metric (Equation (4)) is defined as the patient-level adjusted excess coding intensity using Equation (2) minus the expected values upon further adjustment by facility-level characteristics as per Equation (3):

(4) $$\mathrm{AECI}[i]=\mathrm{ECI}[i]-E(\mathrm{ECI}[i]|\mathrm{FL}[i])=\mathrm{ECI}[i]-\hat{\theta }\times \mathrm{FL}[i]$$

where AECI[i] corresponds to excess coding intensity for patient i further adjusted for facility characteristics of the facility attended by that patient. This new metric encompasses the unexplained (by facility characteristics) variability in the estimated excess coding intensity of patient i. This metric serves to compare facilities by grouping AECI values of all patient visits within each facility. Values of AECI represent the facility-level idiosyncratic variability in coding intensity unexplained by patient- and facility-level variability.

Through this dual-metric approach, we account for both patient-level (using metric ECI) as well as patient- and facility-level (using metric AECI) adjusted comparisons of excess coding intensity across facilities. While the former can be used for comparisons in which facility-level differences in coding intensity are not relevant, the latter can be used to compare differences by facilities in which adjustments by facility-level characteristics are needed.

Incidence rate ratio (IRR) and odds ratio (OR) coefficient estimates and corresponding 95% confidence intervals (CIs) were reported. Root-mean-square errors (RSMEs) were calculated to compare the risk-adjusted model, accounting only for patient characteristics, with a non-informative model represented by the mean level of the response variable.

Negative ECI values represent coding intensity of procedures below average industry standards, with positive values representing over-coding of procedures against these industry standards. An example of stratification for a cohort with equal facility-level characteristics is provided in Supplementary Table S2. Seasonality of AECI was assessed by admission month. A spatial visualization is provided to demonstrate seasonal differences by Census region upon calculating the average AECI per region (AAECI). R version 4.0.3 was used in the analysis.

## 3. Results

### 3.1. Descriptive Statistics

Table 1 provides descriptive statistics for all variables contained in the dataset across 313,477 unique patient visits after collapsing categories with observed frequencies below 0.1%. The outcome variable, additional coded procedures, has a mean of 0.55 (standard deviation; SD 1.05). The mean length of the inpatient stay was 2.47 (SD 2.50) days. Due to its high positive skewness, length of stay was transformed into a logarithmic scale (Supplementary Figures S1 and S2 portray histograms of the length of stay and log of the length of stay, respectively). Nearly 80% of patients were aged 60 years and older (248,364; 79.2%). Most patients identified as female (188,047; 60.0%), White (264,572; 84.4%), mainly attending facilities in urban areas (276,900; 88.3%) with a primary payor status of Medicare Traditional (126,290; 40.3%). The most frequent MS-DRG code was 470, major joint replacement or reattachment of the lower extremity (266,124; 84.9%), with osteoarthritis of the knee (M17) being the most used principal diagnosis code (139,104; 44.4%).

### 3.2. Model Outcomes

We achieved a 21.41% reduction in RMSE (0.83 vs. 1.05) using the zero-inflated Poisson regression model, risk-adjusting by patient-level characteristics, compared to a non-informative model using only the observed mean response without accounting for patient-level characteristics, respectively.

Table 2 contains the zero-inflated Poisson regression IRRs, ORs, and 95% CIs by patient-level characteristics. Each additional unit of the log length of stay is associated with a 43% increase in additional procedure coding incidence rate (IRR = 1.43; 95% CI: 1.41–1.44) after controlling for all other patient characteristics. Pacific Islander patients are associated with a 13% lower incidence rate (IRR = 0.87; 95% CI: 0.79–0.96) of coding additional procedures compared to White patients. When considering the zero-inflation component, those who identify as male have 4.45% higher odds (OR = 1.04; 95% CI: 1.00–1.09) of having zero additional procedures coded than those who identify as female, again controlling for all other patient characteristics. Each additional unit of the AHRQ vulnerability index is associated with 11% increased odds (OR = 1.11; 95% CI: 1.03–1.20) of having zero additional procedures coded.

Table 3 represents results from the multivariate linear regression between excess coding intensity and facility characteristics. A statistically significant difference was found between academic and non-academic hospitals (*p* < 0.0001), with academic facilities coding an average of 0.18 fewer additional procedures per patient visit than non-academic facilities. Furthermore, while controlling for patient and other facility-level characteristics, there is an estimated average difference of 0.03 additional coded procedures per patient visit between urban and rural hospitals, with rural hospitals reporting higher excess coding intensity than urban hospitals (*p* < 0.0001).

Supplementary Table S2 presents results of a multivariate linear regression of excess coding intensity against facility ID for all patient visits of a stratum of facilities with equal facility-level characteristics corresponding to the most observed category for each facility-level variable. Furthermore, Supplementary Figure S3 shows the effect plot of excess coding intensity across facility IDs for this stratum. Although the facilities displayed have the same facility-level features, their excess coding intensity is considerably different, and idiosyncratic characteristics still offer explanatory power of coding differences upon adjusting for facility-level characteristics (*p* < 0.0001). Supplementary Figures S4–S11 portray effect plots of excess coding intensity by the facility-level coefficients.

**Figure 1:**
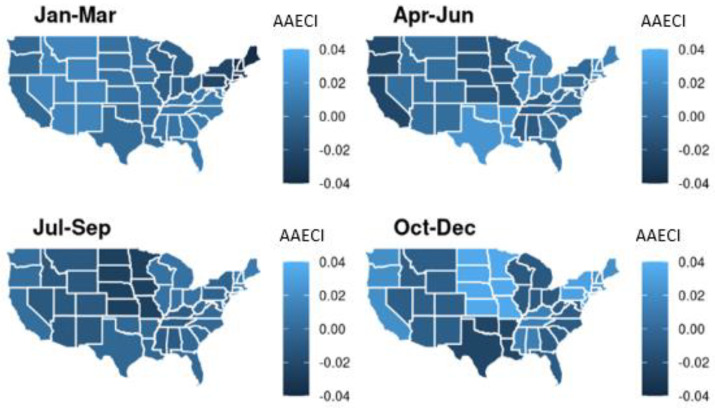
Aggregate adjusted excess coding intensity (AAECI) by quarter and U.S. Census regional division for 2019.

Upon calculating the AECI from the residuals of the ECI regression on facility-level characteristics, we assessed their seasonality by admission month and averaged it within each region based on the U.S. Census Bureau definition of nine regional divisions using a heatmap (Supplementary Figure S12). Lighter shades represent high aggregate AECI (AAECI) and darker shades represent low AAECI. Figure 1 displays the aggregate AAECI by 2019 quarter and U.S. Census regional division, with the lowest AAECI occurring in the New England region during the first quarter of 2019 (January–March; AAECI −0.039), and the highest AAECI observed in the West North Central Census division during the last quarter (October–December; AAECI 0.035). We can also observe inter-division variability of AAECI by quarter, with the last quarter having the greatest inter-region variation in AAECI (−0.023 to 0.035) and the third quarter having the smallest inter-region variation in AAECI (−0.026 to 0.009).

**Figure 2:**
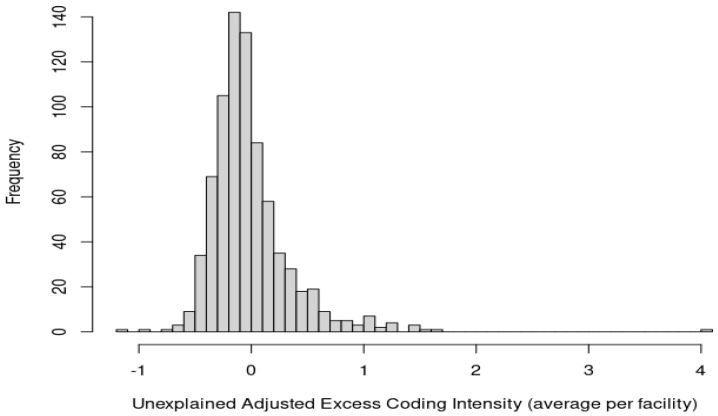
Unexplained adjusted excess coding intensity (AECI) depicted as averages across patient visits per facility for *n* = 781 facilities.

Supplementary Figure S13 displays the effect plot of AECI by facility, and Supplementary Figure S14 visualizes the regression of the adjusted excess coding intensity by facility where coefficients represent the excess coding as a sorted dot plot of the average AECI by facility. Figure 2 portrays the unexplained AECI averaged across patient visits per facility for 781 facilities in the data. Using this visualization and corresponding numeric values, facilities can be ranked—i.e., particularly identify facilities in the tails, whether under-coding or over-coding—that may not be coding similarly to industry standards.

## 4. Discussion

A zero-inflated Poisson model is proposed to estimate excess coding intensity while adjusting for patient-level characteristics, and further adjustment by facility-level characteristics is also proposed on the resulting metric. By using this dual-metric approach, we can identify facilities with potential over- and under-coding when compared to industry standards. Our first proposed metric, a zero-inflated Poisson-derived excess coding intensity estimate, ECI, associated with each patient visit, allows for a view of excess coding intensity adjusted for patient-level characteristics. The second proposed metric, AECI, allows for a two-step approach further adjusting the aforementioned metric by facility-level characteristics. Both metrics allow the user to identify differences in facilities, at the patient-visit level, where potential quality enhancements can be achieved upon adjusting for common sources of variability.

Race was found to be a significant factor, with Pacific Islanders experiencing greater under-coding compared to White patients upon adjusting for other patient-level characteristics. This may be affected by differences in relative regional distribution of the two populations, and the associated differences found when adjusting by U.S. Census regional division. Males experienced high variability in their coding intensity, and were found to be more likely to receive approximately 5% additional coded procedures than women, but they were also found more likely to experience zero excess coding (i.e., no additional coded procedures). This may be associated with higher variability in their underlying clinical conditions. Younger patients were found to experience greater excess coding intensity. Given the nature of the primary procedure (hip/knee arthroplasty), this could be due to larger proportions of non-degenerative underlying causes, such as traumatic and/or urgent procedures, when compared to older patients, with additional procedures needed in parallel to the main procedure. It could also be a result of being healthy enough to undergo multiple procedures at a single visit compared to older and potentially less healthy populations. Length of stay is positively associated with intensity of coding, which is expected, as longer stays are likely to be needed for more complicated clinical cases, thus potentially needing additional procedures. Variability was also found by primary payor, with those self-paying, for example, being less likely to receive additional procedures. This may be voluntary, as self-pay patients may be more likely to reject additional procedures due to cost, or be part of hospital-related processes, where those additional procedures are not offered at the same rate for self-pay patients. Point of origin is also found to be significant, with this variable being a proxy for clinical differences. For example, those transferred from an ambulatory surgery center may be more severe cases and more likely to experience a higher number of procedures than those coming from a non-healthcare facility. Similar conclusions can be reached from discharge status, which may be associated with the severity of the patient’s condition at entry. For example, those transferred to other facilities or to hospice-medical facilities are found to have experienced higher coding intensity than those discharged to home or self-care. Differences in coding intensity can also be expected by underlying ICD-10 principal diagnosis code. For example, those experiencing complications from internal orthopedic prosthetics experience higher coding intensity than those with a more common osteoarthritis of knee (reference category). Similarly, patients with MS-DRGs relating to hip/knee replacement without major complications (reference category) were found to experience lower coding intensity of additional procedures than those in other categories, such as those experiencing complications.

Upon further adjustment for facility-level characteristics, substantial differences were found by academic status of the facility, where academic facilities were found to provide lower adjusted excess coding intensity of procedures than non-academic facilities. Since patient-level differences were already accounted for in the underlying metric, these differences may be due to substantial differences in practice standards when compared to non-academic facilities. However, it is unclear which ones represent a more appropriate standard of care, thus requiring a more qualitative assessment. However, some of these differences are partly offset when accounting for the teaching status of the facility, where teaching facilities provide higher coding intensity to their patients. Physician-led facilities offer lower coding intensity than the more common non-profit-private facilities. This could be due to differences in resources to treat more complex cases, which may be referred to larger facilities with more resources. Other differences were also found by type of government ownership, with those owned by the state coding substantially more than those defined as hospital district or authority. Differences by Census region were also found, with the largest differences between West North Central (lowest excess coding) and West South Central (highest excess coding) with average differences between the two regions of approximately 0.30 procedures per patient visit. The cause of this substantial regional difference between two adjacent regions is unclear. Finally, the patient case mix index of the facility is also a relevant factor, as expected since the case mix index accounts for aggregate patient complexity within the facility. However, it is interesting that facilities with a large case mix index provide lower coding intensity, which may be an indication of stretched facilities with limited resources. Further exploration of this issue is needed, as it may be indicative of under-coding or under-treatment within facilities with large case mix indices.

Admission month is significantly associated with excess coding intensity (*p* = 0.0174). Figure S5 shows the seasonal components of excess coding intensity. Patients admitted for surgery during the month of August experience the lowest excess coding intensity, while patients admitted during the following two months experience the highest levels of excess coding intensity. This may indicate that standards of care are not constant throughout the year, and there may be under-coding occurring during August, a month when some of the permanent or seasoned personnel may schedule vacation time.

While the literature relating to coding intensity of procedures is limited, several similar studies have focused on Current Procedural Terminology (CPT) codes. Haq et al. proposed a deep learning approach for predicting surgery CPTs based on ICD-10 codes input by doctors [25]. ICD-10 and CPT codes are similar yet separate coding systems. While ICD-10 codes capture patient diagnoses across inpatient and ambulatory settings, ICD-10 and CPT procedure codes represent the treatment or procedures provided for such diagnoses across the inpatient, outpatient and ambulatory settings, respectively [26].

In a 2019 national study comparing over 40,000 general emergency department (ED) encounters between academic and non-academic hospitals, Reznek et al. aimed to identify differences in emergency department (ED) clinical operations [27]. More specifically, a secondary scope of the study investigated variation in CPT coding practices among academic and non-academic EDs. It is important to note that this study does not risk-adjust by patient characteristics. While CPT coding may inadequately represent patients with high rates of acute diagnosis, Reznek et al. found no significant association in CPT coding intensity between academic and non-academic facilities [27]. The aforementioned findings do not align with the included analysis, which identified significant differences in ICD-10 coding intensity by academic status, with academic facilities coding fewer additional procedures per patient in contrast to non-academic facilities. However, our study findings account and adjust for patient-level differences, which can be a major confounder when comparing results by facility due to differences in patient mix.

### Strengths and Limitations

We assess the outcome (additional recorded procedure counts) while addressing a large number of patient visits with 0 counts, thus lending itself to a zero-inflated Poisson model. A flexible model that accounts for zero inflation is needed to account for cases where no additional procedures are explored (possible under-treatment) or coded (under-coding) regardless of potential need, which may occur at different levels for different cohorts/procedures. While regular zero counts can be captured by a regular Poisson, zero-inflation offers the flexibility of accounting for excess zeros unexplained by the underlying expected excess procedure rates. Using administrative claims data, our approach can assess whether facilities had a positive or negative excess coding of procedures by controlling for the patient- and facility-level characteristics. This study fills a substantial gap in the current literature by providing a measure at the patient-level for the expected coding intensity for procedures. This measure can be aggregated across different dimensions, such as hospitals and physicians, and differences can be explored at different levels, such as by seasonal component or its evolution over time, to assess quality improvements.

A limitation with using patient-level characteristics from claims data is the lack of complete information about the severity of the patient’s health and other underlying reasons for additional procedures, which are not available without clinical information or health records. Since the severity is not explicitly stated, nor whether the procedure was elective or urgent, we did not control for the association it may have on additional procedures. However, departures from common industry practice, when averaged across patients and upon adjusting for case mix index, can provide a glimpse at facilities that may be delivering healthcare in different forms relative to industry standards, leading to possible under- or over-coding practices.

Multicollinearity is present among some covariates, so a cautious approach is recommended when interpreting the variable relevance during the risk adjustment. However, this does not affect the core outcomes of our approach, which are the ECI and AECI metrics extracted through the corresponding model residuals. These two metrics (or a combined metric if we wanted to incorporate the facility-level characteristics as part of the zero-inflated model), can be defined in the presence of multicollinearity, which is common during risk adjustment.

We define a two-step approach for incorporating the patient-level and facility-level characteristics, with different metrics defined after sequentially risk-adjusting for each. This allows for various uses, as ECI can be applied when facility-level risk adjustments are not appropriate (i.e., when providing payments for services) and AECI can be utilized when they are (i.e., when resources or expectations are not equal across providers). However, a combined risk adjustment within the zero-inflated Poisson model is possible. This does not invalidate the approach, as the objective is to demonstrate how metrics can be defined for various types of potential risk adjustments; however, the risk-adjusting factors can be user-defined based on preferences and desired metric outcomes. Additionally, while our approach does not use patient-level clinical data and is solely based on claims data, clinical information could easily be added as additional covariates, if available.

Although outside the scope of our study, the proposed method lends itself to a temporal analysis of facility performance. The proposed metrics can be reproduced over time, and relative and absolute facility excess coding intensity (through ECI or AECI) could be monitored dynamically both against itself and against peer performance. This can serve to identify needs and monitor efforts in quality improvement.

While we define the model against industry standards, defined as overall industry averages, a reference group of hospitals can be used to define a benchmark, if they can be identified as industry leaders in standards of care. Those hospitals can serve as references for defining model coefficients, and other hospitals can be measured against those benchmarking references.

Finally, the proposed approach can be generalizable and is not dependent on the availability of clinical patient data, which can be heavily sparse. Additionally, by not relying on clinical thresholds or markers, it can be adapted to other cohorts and automatized across them, producing metrics that can be used easily by quality control practitioners across a wide variety of health conditions. This can further serve to monitor overall standards by facility, where differences in standards of practice may be more obscure within a particular cohort, but become clearer when measured across cohorts and conditions.

## 5. Conclusions

Our model is generalizable to other patient cohorts, computationally easy to implement, and does not rely on clinical information or patient medical records. The outcomes include tools to identify facilities that may be operating differently from their peers or industry benchmarks. Substantial differences were found in procedure coding intensity at all levels (patient visit, facility, time, and region). Our metrics do not identify differences in quality but instead include potential differences in processes that could be modified (or adopted by others) and create the opportunity for quality assessment, control, and enhancement regarding procedure coding intensity.

## Figures and Tables

**Table 1:** Descriptive statistics (count/mean and percentage/standard deviation, respectively) of patient characteristics, facility characteristics, and the outcome (additional coded procedures) for *n* = 313,477 unique patient visits.

Variable	Count/Mean (%/SD)
Additional Coded Procedures (Outcome)	0.55 (1.05)
Length of Stay (days)	2.47 (2.50)
AHRQ ^1^ Overall Tract Summary	0.50 (0.25)
Patient Age Group	
<25	235 (0.07%)
25–34	983 (0.31%)
35–44	4598 (1.47%)
45–54	25,608 (8.17%)
55–59	33,689 (10.75%)
60–64	47,628 (15.19%)
65–69	57,813 (18.44%)
70–74	54,614 (17.42%)
75–79	41,090 (13.11%)
80–84	25,633 (8.18%)
>84	21,586 (6.89%)
Patient Sex	
Female	188,047 (59.99%)
Male	125,391 (40.00%)
Unknown	39 (0.01%)
Patient Race	
American Indian	1235 (0.39%)
Asian	3532 (1.13%)
Black	26,053 (8.31%)
Pacific Islander	1061 (0.34%)
White	264,572 (84.40%)
Other	11,934 (3.81%)
Unable to Determine	5090 (1.62%)
Primary Payor	
Commercial Indemnity	24,399 (7.78%)
Direct Employer Contract	1125 (0.36%)
Managed Care Capitated	878 (0.28%)
Managed Care Non-Capitated	62,892 (20.06%)
Medicaid Managed Care Capitated	1571 (0.50%)
Medicaid Managed Care Non-Capitated	10,231 (3.26%)
Medicaid Traditional	3020 (0.96%)
Medicare Managed Care Capitated	11,770 (3.75%)
Medicare Managed Care Non-Capitated	58,456 (18.65%)
Medicare Traditional	126,290 (40.29%)
Other Government Payors	4983 (1.59%)
Self-Pay	1295 (0.41%)
Workers Compensation	2703 (0.86%)
Other	3864 (1.23%)
Source of Admission	
Clinic	81,515 (26.00%)
Non-Healthcare Facility (Physician Referral)	223,009 (71.14%)
Transfer from a Hospital (Different Facility)	3811 (1.22%)
Transfer from Ambulatory Surgical Center	373 (0.12%)
Transfer from Another Healthcare Facility	1630 (0.52%)
Transfer from SNF ^2^ or ICF ^3^	1631 (0.52%)
Other	280 (0.09%)
Information Not Available	1228 (0.39%)
Patient Discharge Status	
Discharged To Home Health Organization	122,915 (39.21%)
Discharged To Home or Self-Care	118,867 (37.92%)
Discharged To Hospice-Medical Facility	385 (0.12%)
Discharged/Transferred to ICF ^3^	316 (0.10%)
Discharged/Transferred to Other Facility	704 (0.22%)
Discharged/Transferred to SNF ^2^	54,977 (17.54%)
Discharged/Transferred to Swing Bed	1515 (0.48%)
Discharged/Transferred to Another Rehabilitation Facility	11,437 (3.65%)
Expired	579 (0.18%)
Other	1782 (0.57%)
ICD-10 Principal Diagnosis Code	
Osteoarthritis of Hip (M16)	104,950 (33.48%)
Osteoarthritis of the Knee (M17)	139,104 (44.37%)
Other and Unspecified Osteoarthritis (M19)	1405 (0.45%)
Osteoporosis with Current Pathological Fracture (M80)	1716 (0.55%)
Disorder of Continuity of Bone (M84)	1044 (0.33%)
Osteonecrosis (M87)	4449 (1.42%)
Periprosthetic Fracture around an Internal Prosthetic Joint (M97)	849 (0.27%)
Fracture of Femur (S72)	31,864 (10.16%)
Complications of Internal Orthopedic Prosthetic Devices, Implants and Grafts (T84)	24,034 (7.67%)
Orthopedic Aftercare (Z47)	1461 (0.47%)
Other and Unspecified Arthropathy (M12)	345 (0.11%)
Other	2256 (0.72%)
Admission Month	
January	29,509 (9.41%)
February	26,548 (8.47%)
March	25,632 (8.18%)
April	27,927 (8.91%)
May	25,914 (8.27%)
June	25,321 (8.08%)
July	26,267 (8.38%)
August	24,262 (7.74%)
September	24,397 (7.78%)
October	28,666 (9.14%)
November	24,669 (7.87%)
December	24,365 (7.77%)
MS-DRG ^4^ Code	
Bilateral or Multiple Major Joint Procedures of Lower Extremity with MCC ^5^ (461)	144 (0.05%)
Bilateral or Multiple Major Joint Procedures of Lower Extremity without MCC ^5^ (462)	5536 (1.77%)
Revision of Hip or Knee Replacement with MCC ^5^ (466)	2838 (0.91%)
Revision of Hip or Knee Replacement with CC ^6^ (467)	13,522 (4.31%)
Revision of Hip or Knee Replacement without CC ^6^/MCC ^5^ (468)	11,533 (3.68%)
Major Hip and Knee Joint Replacement or Reattachment of Lower Extremity with MCC ^5^ or Total Ankle Replacement (469)	13,780 (4.40%)
Major Joint Replacement or Reattachment of Lower Extremity Without MCC ^5^ (470)	266,124 (84.89%)
Facility Case Mix Index	
0	12,173 (3.88%)
1	103,626 (33.06%)
2	196,196 (62.59%)
3	1482 (0.47%)
Facility Teaching Status	
No	247,890 (79.08%)
Yes	60,360 (19.26%)
TBD	5227 (1.67%)
Facility Academic Status	
No	274,759 (87.65%)
Yes	38,718 (12.35%)
Facility Urban/Rural Status	
Rural	36,577 (11.67%)
Urban	276,900 (88.33%)
Facility Ownership Status	
Government–Federal	354 (0.11%)
Government–Hospital District or Authority	15,441 (4.93%)
Government–Local	6458 (2.06%)
Government–State	2014 (0.64%)
Physician	1359 (0.43%)
Proprietary	13,924 (4.44%)
Voluntary Non-Profit–Church	45,070 (14.38%)
Voluntary Non-Profit–Private	211,855 (67.58%)
Voluntary Non-Profit–Other	17,002 (5.42%)
Facility Bed Count	
1–100	28,147 (8.98%)
101–200	56,860 (18.14%)
201–300	65,391 (20.86%)
301–400	51,761 (16.51%)
401–500	36,550 (11.66%)
501–600	27,249 (8.69%)
601–700	17,373 (5.54%)
701–800	13,822 (4.41%)
801–900	8938 (2.85%)
901–1000	3267 (1.04%)
1001–2000	4119 (1.31%)
Census Regional Division	
East North Central	59,839 (19.09%)
East South Central	26,505 (8.46%)
Middle Atlantic	44,215 (14.10%)
Mountain	15,533 (4.96%)
New England	13,824 (4.41%)
Pacific	24,880 (7.94%)
South Atlantic	79,718 (25.43%)
West North Central	22,444 (7.16%)
West South Central	26,519 (8.46%)

^1^ AHRQ: Agency for Healthcare Research and Quality. ^2^ SNF: Skilled nursing facility. ^3^ ICF: Intermediate care facility. ^4^ MS-DRG: Medicare Severity Diagnosis Related Groups. ^5^ MCC: Major Complication or Comorbidity. ^6^ CC: Complication or Comorbidity.

**Table 2:** Zero-inflated Poisson regression incidence rate ratios (IRRs) and odds ratios (ORs), and corresponding 95% confidence intervals (CIs), for additional procedure counts.

	IRR	95% CI	OR	95% CI
Intercept	0.46	0.44–0.47	0.46	0.42–0.50
Log (Length of Stay)	1.43	1.41–1.44	1	0.96–1.04
AHRQ ^1^ Overall Tract Summary	0.9	0.88–0.92	1.11	1.03–1.20
Age Group				
<25	1.35	1.16–1.57	0.54	0.26–1.11
25–34	1.3	1.19–1.42	0.75	0.53–1.06
35–44	1.21	1.15–1.27	0.72	0.60–0.87
45–54	1.13	1.09–1.16	0.83	0.74–0.93
55–59	1.11	1.07–1.14	0.91	0.82–1.00
60–64	1.09	1.06–1.12	0.86	0.78–0.95
65–69	1.09	1.06–1.12	0.92	0.85–1.00
70–74	1.06	1.03–1.09	0.93	0.86–1.01
75–79	1.04	1.01–1.07	0.92	0.85–0.99
80–84	1.01	0.98–1.04	0.92	0.85–1.00
Sex				
Male	1.05	1.04–1.06	1.04	1.00–1.09
Unknown	1.28	0.82–2.01	2.24	0.64–7.90
Race				
American Indian	1.02	0.93–1.11	1.01	0.75–1.36
Asian	0.99	0.93–1.05	1.08	0.90–1.30
Black	1	0.97–1.02	0.97	0.90–1.04
Pacific Islander	0.87	0.79–0.96	1.16	0.81–1.66
Other	0.99	0.96–1.02	1.01	0.91–1.13
Unable To Determine	0.98	0.93–1.03	0.93	0.79–1.09
Primary Payor				
Commercial–Indemnity	0.99	0.96–1.01	0.86	0.78–0.95
Direct Employer Contract	0.95	0.85–1.07	1.27	0.90–1.80
Managed Care–Capitated	1.11	1.00–1.23	1.41	1.03–1.93
Managed Care–Non-Capitated	0.97	0.95–1.00	1.07	1.00–1.15
Medicaid–Managed Care Capitated	0.95	0.88–1.03	0.68	0.49–0.94
Medicaid–Managed Care Non-Capitated	0.92	0.89–0.95	0.95	0.84–1.07
Medicaid–Traditional	0.97	0.92–1.02	1.32	1.10–1.59
Medicare–Managed Care Capitated	0.95	0.92–0.98	0.82	0.74–0.92
Medicare–Managed Care Non-Capitated	0.96	0.95–0.98	1.15	1.09–1.21
Other Government Payors	0.92	0.87–0.96	0.91	0.77–1.09
Self-Pay	0.88	0.80–0.97	0.9	0.66–1.21
Workers Compensation	0.9	0.85–0.95	0.58	0.44–0.77
Other	0.92	0.87–0.98	0.79	0.65–0.97
Point of Origin				
Clinic	0.92	0.91–0.94	1.25	1.19–1.32
Information Not Available	1.14	1.05–1.25	1.31	1.03–1.66
Other	0.76	0.64–0.90	2.2	1.31–3.69
Transfer From a Hospital (Different Facility)	1.04	1.00–1.07	0.93	0.83–1.06
Transfer From Ambulatory Surgery Center	1.24	1.05–1.47	3.41	2.25–5.16
Transfer From Health Facility	0.97	0.91–1.04	1.12	0.88–1.42
Transfer From SNF ^2^ Or ICF ^3^	0.99	0.94–1.06	1.03	0.87–1.23
Discharge Status				
Discharged To Home Health Organization	1.01	0.99–1.02	0.74	0.71–0.78
Discharged To Hospice-Medical Facility	1.17	1.07–1.29	0.67	0.50–0.89
Discharged/Transferred to ICF ^3^	1.02	0.86–1.21	1.49	0.99–2.26
Discharged/Transferred to Other Facility	1.27	1.18–1.37	0.62	0.44–0.86
Discharged/Transferred to SNF ^2^	0.95	0.93–0.97	0.75	0.70–0.81
Discharged/Transferred to Swing Bed	0.99	0.92–1.06	0.6	0.47–0.77
Discharged/Transferred to Another Rehab Facility	1.03	1.00–1.06	0.71	0.64–0.78
Expired	2.19	2.09–2.30	0.22	0.17–0.29
Other	1.22	1.16–1.28	0.73	0.61–0.89
ICD-10 Principal Diagnosis Code				
Other and Unspecified Arthropathy (M12)	1.26	1.13–1.41	0	0.00–Inf ^7^
Osteoarthritis of Hip (M16)	0.85	0.82–0.87	2.81	2.64–3.00
Other and Unspecified Osteoarthritis (M19)	1.34	1.25–1.44	0.45	0.32–0.65
Osteoporosis with Current Pathological Fracture (M80)	1.04	0.96–1.13	3.31	2.76–3.97
Disorder of Continuity of Bone (M84)	1.37	1.28–1.47	1.29	1.02–1.64
Osteonecrosis (M87)	0.92	0.85–1.00	1.85	1.52–2.25
Periprosthetic Fracture around Internal Prosthetic Joint (M97)	1.15	1.09–1.22	0.03	0.00–Inf ^7^
Fracture of Femur (S72)	1.04	1.01–1.08	3.24	2.97–3.53
Complications of Internal Orthopedic Prosthetic Devices, Implants and Grafts (T84)	1.34	1.30–1.38	0	0.00–Inf ^7^
Orthopedic Aftercare (Z47)	1.08	1.02–1.13	0.47	0.11–2.05
Other	1.39	1.33–1.46	0.69	0.52–0.92
MS-DRG ^4^ Code				
Bilateral or Multiple Major Joint Procedures of Lower Extremity with MCC ^5^ (461)	3.39	3.10–3.72	0	0.00–Inf ^7^
Bilateral or Multiple Major Joint Procedures of Lower Extremity without MCC ^5^ (462)	2.29	2.23–2.35	0	0.00–Inf ^7^
Revision of Hip or Knee Replacement with MCC ^5^ (466)	3.05	2.95–3.16	0	0.00–Inf ^7^
Revision of Hip or Knee Replacement with CC ^6^ (467)	2.51	2.44–2.58	0	0.00–Inf ^7^
Revision of Hip or Knee Replacement without CC ^6^/MCC ^5^ (468)	2.26	2.20–2.34	0	0.00–Inf ^7^
Major Hip and Knee Joint Replacement or Reattachment of Lower Extremity with MCC ^5^ or Total Ankle Replacement (469)	2.02	1.96–2.08	0.79	0.74–0.85

^1^ AHRQ: Agency for Healthcare Research and Quality. ^2^ SNF: Skilled nursing facility. ^3^ ICF: Intermediate care facility. ^4^ MS-DRG: Medicare Severity Diagnosis Related Groups. ^5^ MCC: Major Complication or Comorbidity. ^6^ CC: Complication or Comorbidity. ^7^ Inf indicates that the value is larger than reportable within computational limits. Reference groups for categorical covariates include the following: White (race); female (sex); M17 (ICD-10 principal diagnosis code); South Atlantic (Census region); 470 (MS-DRG); over 84 (age); Medicare–Traditional (primary payor); non-healthcare facility (point of origin); discharged to home or self-care (discharge status).

**Table 3:** Summary of the multivariate linear regression between excess coding intensity and facility characteristics.

	Estimate	Std. Error	*p*-Value
Intercept	−0.086	0.01	<0.0001
Teaching Status			
Yes	0.104	0.006	<0.0001
TBD	0.032	0.012	0.0074
Academic Status			
Yes	−0.183	0.008	<0.0001
Urban/Rural Status			
Rural	0.028	0.005	<0.0001
Ownership Status			
Government–Federal	−0.026	0.044	0.5633
Government–Hospital District or Authority	−0.144	0.007	<0.0001
Government–Local	−0.064	0.011	<0.0001
Government–State	0.082	0.019	<0.0001
Physician	−0.121	0.023	<0.0001
Proprietary	0.01	0.008	0.1903
Voluntary Non-Profit–Church	0.064	0.005	<0.0001
Voluntary Non-Profit–Other	0.017	0.007	0.0135
Bed Count			
100–200	0.011	0.006	0.0819
201–300	0.091	0.006	<0.0001
301–400	0.086	0.007	<0.0001
401–500	0.075	0.007	<0.0001
501–600	−0.023	0.008	0.0024
601–700	0.123	0.009	<0.0001
701–800	0.129	0.01	<0.0001
801–900	0.196	0.012	<0.0001
901–1000	−0.001	0.027	0.9672
1001–2000	0.018	0.015	0.2122
Census Regional Division			
East North Central	0.095	0.005	<0.0001
East South Central	−0.069	0.006	<0.0001
Middle Atlantic	−0.035	0.005	<0.0001
Mountain	−0.046	0.007	<0.0001
New England	0.038	0.008	<0.0001
Pacific	0.007	0.006	0.2489
West North Central	−0.098	0.006	<0.0001
West South Central	0.191	0.006	<0.0001
Case Mix Index			
1	0	0.008	0.7408
2	0.01	0.008	0.1865
3	−0.24	0.025	<0.0001

Reference groups for categorical covariates include the following: no (teaching status); no (academic status); urban (urban/rural status); voluntary non-profit–private (ownership status); 0–100 (bed count); South Atlantic (Census regional division); and 0 (case mix index).

## Data Availability

Data was provided by Premier, Inc., and is proprietary but accessible for purchase.

## Supplementary Materials

The following supporting information can be downloaded at: https://www.mdpi.com/article/10.3390/healthcare10081368/s1, Table S1: MS-DRG codes related to hip/knee arthroplasty procedures; Table S2: Results of a multivariate linear regression of excess coding intensity against facility ID for all patient visits of a stratum of facilities with equal facility-level characteristics (*n* = 2058 patient visits); Figure S1: Histogram of length of stay (days); Figure S2: Histogram of log length of stay; Figure S3: Effect plot of excess coding intensity by facility ID for facilities that share the same facility-level characteristics; Figure S4: Effect plot of excess coding intensity by admission month; Figure S5: Effect plot of excess coding intensity by facility urban/rural status; Figure S6: Effect plot of excess coding intensity by facility ownership; Figure S7: Effect plot of excess coding intensity by facility case mix index (CMI); Figure S8: Effect plot of excess coding intensity by facility teaching status; Figure S9: Effect plot of excess coding intensity by facility academic status; Figure S10: Effect plot of excess coding intensity by facility U.S. Census regional division; Figure S11: Effect plot of excess coding intensity by facility bed count; Figure S12: Heatmap of AECI seasonality by admission month and facility region based on the U.S. Census Bureau definition of 9 regional divisions; Figure S13: Effect plot of adjusted excess coding intensity (AECI) by facility; Figure S14: Sorted dot plot of adjusted excess coding intensity (AECI) across facilities.

## References

[1] World Health Organization International Statistical Classification of Diseases and Related Health Problems (ICD). https://www.who.int/standards/classifications/classification-of-diseases.

[2] Storesund A., Haugen A.S., Hjortås M., Nortvedt M.W., Flaatten H., Eide G.E., Boermeester M.A., Sevdalis N., Søfteland E. (2019). Accuracy of surgical complication rate estimation using ICD-10 codes. Br. J. Surg..

[3] Misset B., Nakache D., Vesin A., Darmon M., Garrouste-Orgeas M., Mourvillier B., Adrie C., Pease S., de Beauregard M.A.C., Goldgran-Toledano D. (2008). Reliability of diagnostic coding in intensive care patients. Crit. Care.

[4] U.S. (2009). Centers for Medicare and Medicaid Services. Acute Inpatient Prospective Payment Systems. https://www.ahd.com/AcutePaymtSysfctsht_JAN09.pdf.

[5] Kronick R. (2017). Projected Coding Intensity In Medicare Advantage Could Increase Medicare Spending By $200 Billion Over Ten Years. Health Aff..

[6] Krumholz H.M., Normand S.T., Galuhsa D.H., Mattera J.A., Rich A.S., Wang Y., Wang Y. Risk-Adjustment Models For AMI and HF 30-day Mortality: Methodology. https://qualitynet.cms.gov/inpatient/measures/mortality/methodology.

[7] Agency for Healthcare Research and Quality Patient Safety Indicators (PSI) Parameter Estimates, v2021. July 2021. https://qualityindicators.ahrq.gov/Downloads/Modules/PSI/V2021/Parameter_Estimates_PSI_v2021.pdf.

[8] Olmsted M.G., Powell R., Murphey J., Bell D., Silver B., Stanley M., Sanchez R.T., Allen R. Methodology: U.S News & World Report 2021-22 Best Hospitals: Specialty Rankings. 27 July 2021. https://health.usnews.com/media/best-hospitals/BH_Methodology_2021-22.

[9] Kronick R., Welch W.P. (2014). Measuring coding intensity in the Medicare Advantage program. Med. Med. Res. Rev..

[10] Henderson T., Shepheard J., Sundararajan V. (2006). Quality of Diagnosis and Procedure Coding in ICD-10 Administrative Data. Med. Care.

[11] Ayub S., Scali S.T., Richter J., Huber T.S., Beck A.W., Fatima J., Berceli S.A., Upchurch G.R., Arnaoutakis D., Back M.R. (2019). Financial implications of coding inaccuracies in patients undergoing elective endovascular abdominal aortic aneurysm repair. J. Vasc. Surg..

[12] Murphy J., May C., Di Carlo S., Beckingham I., Cameron I.C., Gomez D. (2018). Coding in surgery: Impact of a specialized coding proforma in hepato-pancreato-biliary surgery. ANZ J. Surg..

[13] Kahn K. Steps to Avoid Overcoding and Undercoding. https://physicians.dukehealth.org/articles/steps-avoid-overcoding-and-undercoding.

[14] Geruso M., Layton T. (2020). Upcoding: Evidence from Medicare on Squishy Risk Adjustment. J. Polit. Econ..

[15] North Carolina Department of Justice Health Fraud. https://ncdoj.gov/responding-to-crime/health-fraud/.

[16] Gold R.S. (2008). Know critical care billing, documentation requirements: Don’t put your career on the line because of fraudulent reporting, overcoding. Med. Rec. Brief..

[17] Beveridge R.A., Mendes S.M., Caplan A., Rogstad T.L., Olson V., Williams M.C., McRae J.M., Vargas S. (2017). Mortality Differences Between Traditional Medicare and Medicare Advantage: A Risk-Adjusted Assessment Using Claims Data. Inquiry.

[18] Clark D.E., Fitzgerald T.L., Dibbins A.W. (2018). Procedure-based postoperative risk prediction using NSQIP data. J. Surg. Res..

[19] Baum G.R., Stricsek G., Kumarasamy M.A., Thirunavu V., Esper G.J., Boden S.D., Refai D. (2021). Current Procedural Terminology-based Procedure Categorization Enhances Cost Prediction of Medicare Severity Diagnosis Related Group in Spine Surgery. Spine.

[20] Kurtz S., Ong K., Lau E., Mowat F., Halpern M. (2007). Projections of Primary and Revision Hip and Knee Arthroplasty in the United States from 2005 to 2030. J. Bone Jt. Surg. Am..

[21] Clair A.J., Inneh I.A., Iorio R., Berend K.R., Della Valle C.J., Healy W.L., Pelligrini V.D. (2015). Can Administrative Data Be Used to Analyze Complications Following Total Joint Arthroplasty?. J. Arthroplast..

[22] Cahue S.R., Etkin C.D., Stryker L.S., Voss F.R. (2019). Procedure coding in the American Joint Replacement Registry. Arthroplast. Today.

[23] Premier Applied Sciences (2020). Premier Healthcare Database White Paper: Data That Informs and Performs.

[24] U.S. Centers for Medicare & Medicaid Services. DRG Classifications and Software. https://www.cms.gov/Medicare/Medicare-Fee-for-Service-Payment/AcuteInpatientPPS/MS-DRG-Classifications-and-Software.

[25] Haq H.U., Ahmad R., Hussain S.U. (2017). Intelligent EHRs: Predicting Procedure Codes From Diagnosis Codes. Machine Learning for Health Workshop at Neural Information Processing Systems.

[26] National Athletic Trainers’ Association Commonly Used CPT Codes. https://www.nata.org/practice-patient-care/revenue-reimbursement/general-revenue-reimbursement/commonly-used-cpt-codes.

[27] Reznek M.A., Michael S.S., Harbertson C.A., Scheulen J.J., Augustine J.J. (2019). Clinical Operations of Academic versus Non-Academic Emergency Departments: A Descriptive Comparison of Two Large Emergency Department Operations Surveys. BMC Emerg. Med..

