# Supplementary material for: Modeling Coding Intensity of Procedures in a U.S. Population-Based Hip/Knee Arthroplasty Inpatient Cohort Adjusting for Patient- and Facility-Level Characteristics
Source: Healthcare (Basel). 2022 Jul 23;10(8):1368. doi: 10.3390/healthcare10081368 (PMC9332158; doi:10.3390/healthcare10081368)

SUPPLEMENTARY MATERIALS

Table S1	MS-DRG codes related to hip/knee arthroplasty procedures	2
Table S2	Results of a multivariate linear regression of excess coding intensity against facility ID for all patient-visits of a stratum of facilities with equal facility-level characteristics (N= 2,058 patient-visits)	3
Figure S1	Histogram of length of stay (days)	4
Figure S2	Histogram of log length of stay	5
Figure S3	Effect plot of excess coding intensity by facility ID for facilities that share the same facility-level characteristics	6
Figure S4	Effect plot of excess coding intensity by admission month	7
Figure S5	Effect plot of excess coding intensity by facility urban/rural status	8
Figure S6	Effect plot of excess coding intensity by facility ownership	9
Figure S7	Effect plot of excess coding intensity by facility case mix index (CMI)	10
Figure S8	Effect plot of excess coding intensity by facility teaching status	11
Figure S9	Effect plot of excess coding intensity by facility academic status	12
Figure S10	Effect plot of excess coding intensity by facility U.S. Census regional division	13
Figure S11	Effect plot of excess coding intensity by facility bed count	14
Figure S12	Heatmap of AECI seasonality by admission month and facility region based on the U.S. Census Bureau definition of 9 regional divisions	15
Figure S13	Effect plot of adjusted excess coding intensity (AECI) by facility	16
Figure S14	Sorted dot plot of adjusted excess coding intensity (AECI) across facilities	17

Table S1: MS-DRG codes related to hip/knee arthroplasty procedures.

MS-DRG Code	MS-DRG Description
461	Bilateral or Multiple Major Joint Procedures of Lower Extremity with Major Complication or Comorbidity (MCC)
462	Bilateral or Multiple Major Joint Procedures of Lower Extremity without MCC
466	Revision of Hip or Knee Replacement with MCC
467	Revision of Hip or Knee Replacement with CC
468	Revision of Hip or Knee Replacement without CC/MCC
469	Major Hip and Knee Joint Replacement or Reattachment of Lower Extremity with MCC or Total Ankle Replacement
470	Major Hip and Knee Joint Replacement or Reattachment of Lower Extremity without MCC

Table S2. Results of a multivariate linear regression of excess coding intensity against facility ID for all patient-visits of a stratum of facilities with equal facility-level characteristics (N= 2,058 patient-visits)

Coefficients	Estimate	Std. Error	t statistic	p-value
(Intercept)	-0.15	0.03	-4.65	<0.0001
Facility ID 115	-0.12	0.04	-2.80	0.0051
Facility ID 290	0.12	0.06	2.08	0.0378
Facility ID 671	0.56	0.04	14.00	< 0.0001
Residual standard error: 0.6848 on 2054 degrees of freedom				
Multiple R-squared: 0.162				

Reference group: Facility ID 78. Common values of facility characteristics: 2 (CMI); No (teaching status); No (academic status); Urban (urban/rural status); Voluntary Non-profit - Private (ownership status); 0-100 (bed count); South Atlantic (Census regional division).

Figure S1. Histogram of length of stay (days)

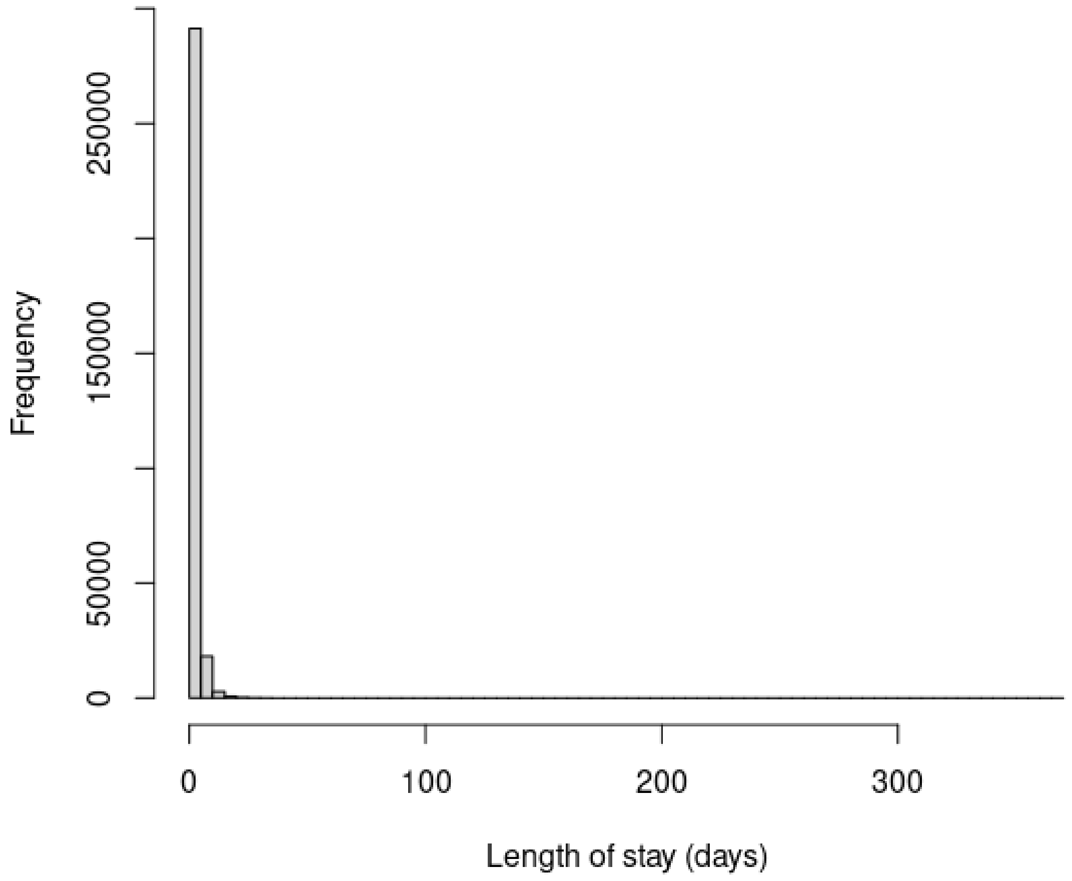

Figure S2. Histogram of log length of stay

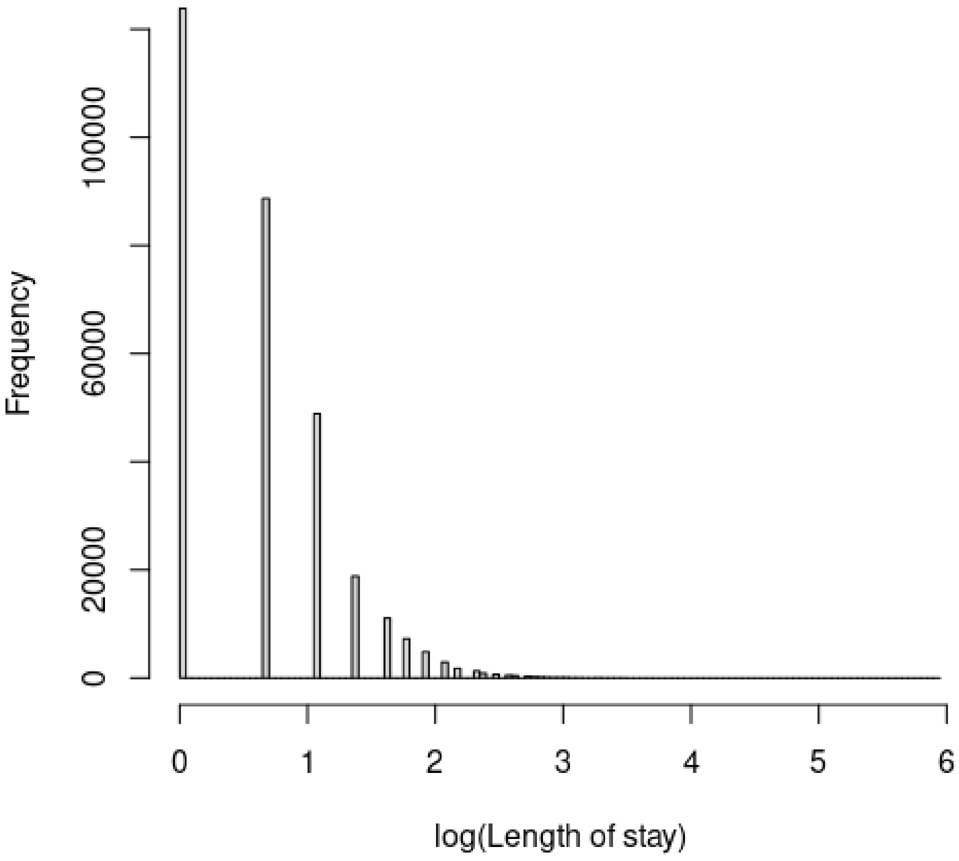

Figure S3. Effect plot of excess coding intensity by facility ID for facilities that share the same facility-level characteristics

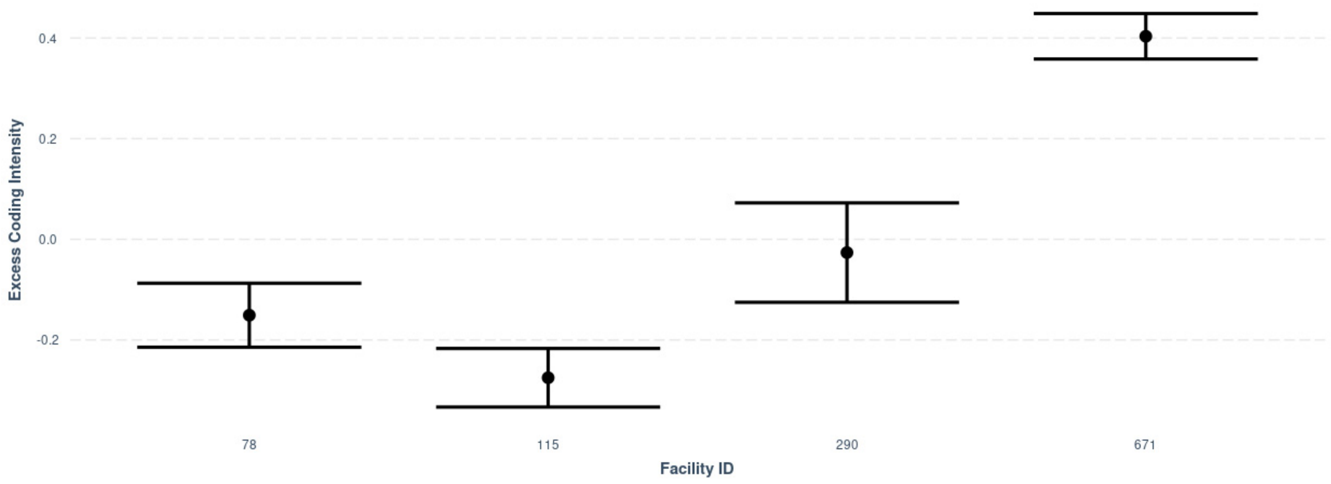

Figure S4. Effect plot of excess coding intensity by admission month

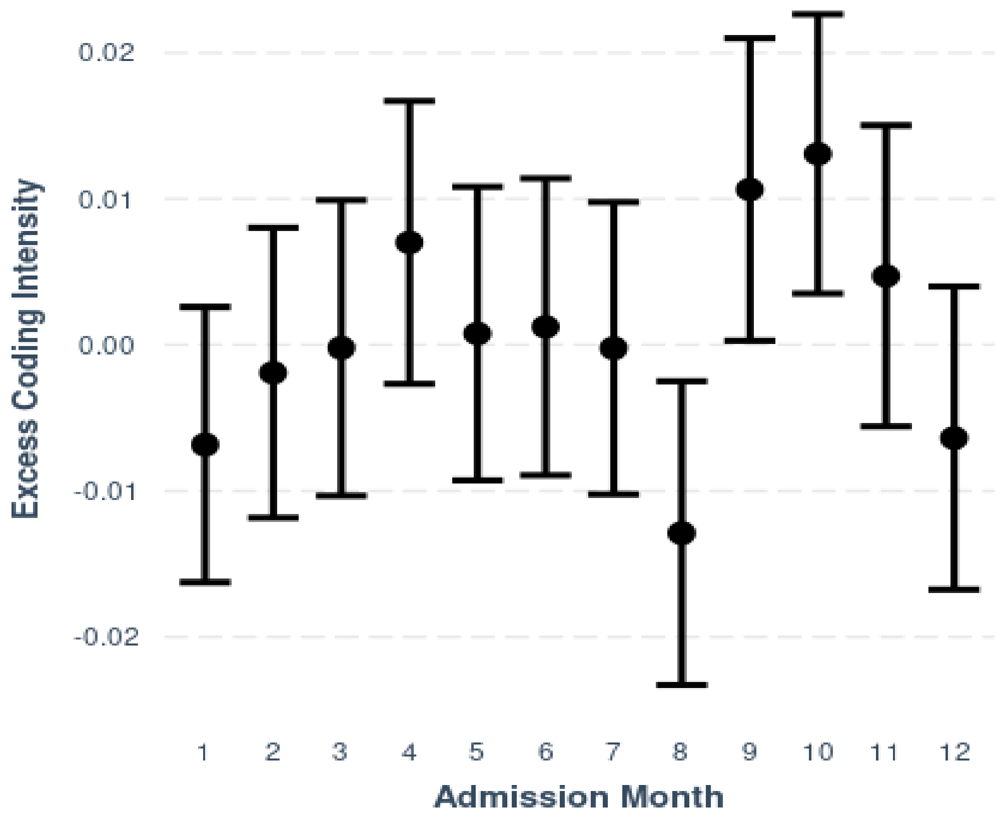

Figure S5. Effect plot of excess coding intensity by facility urban/rural status

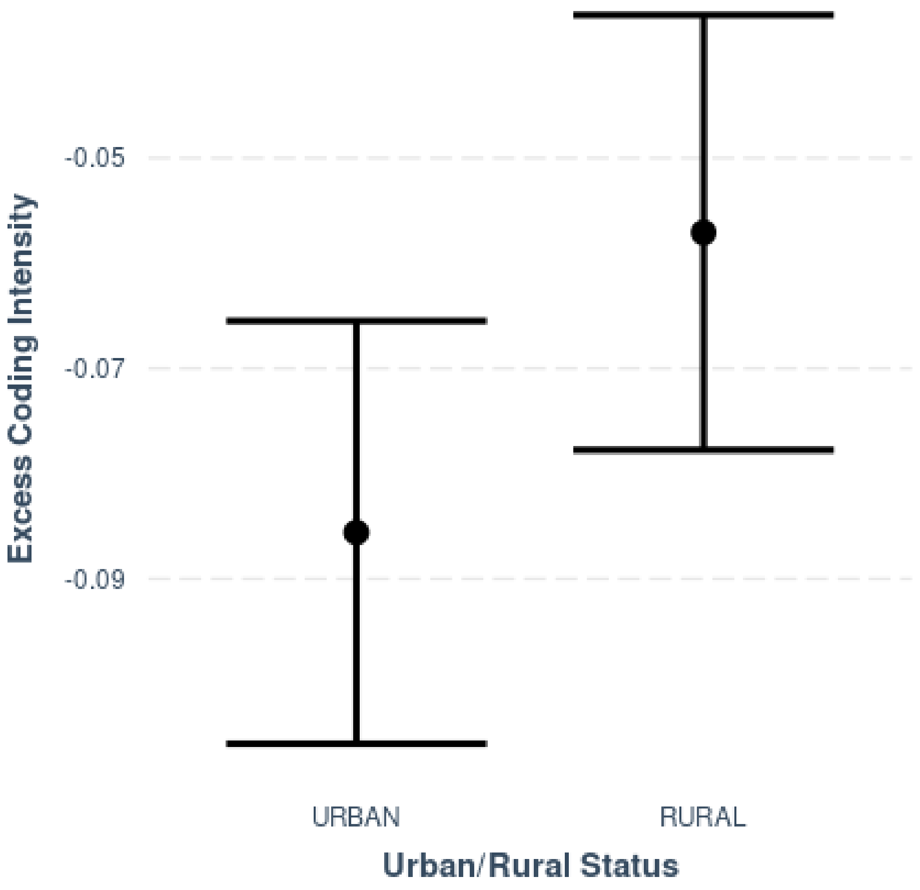

Figure S6. Effect plot of excess coding intensity by facility ownership

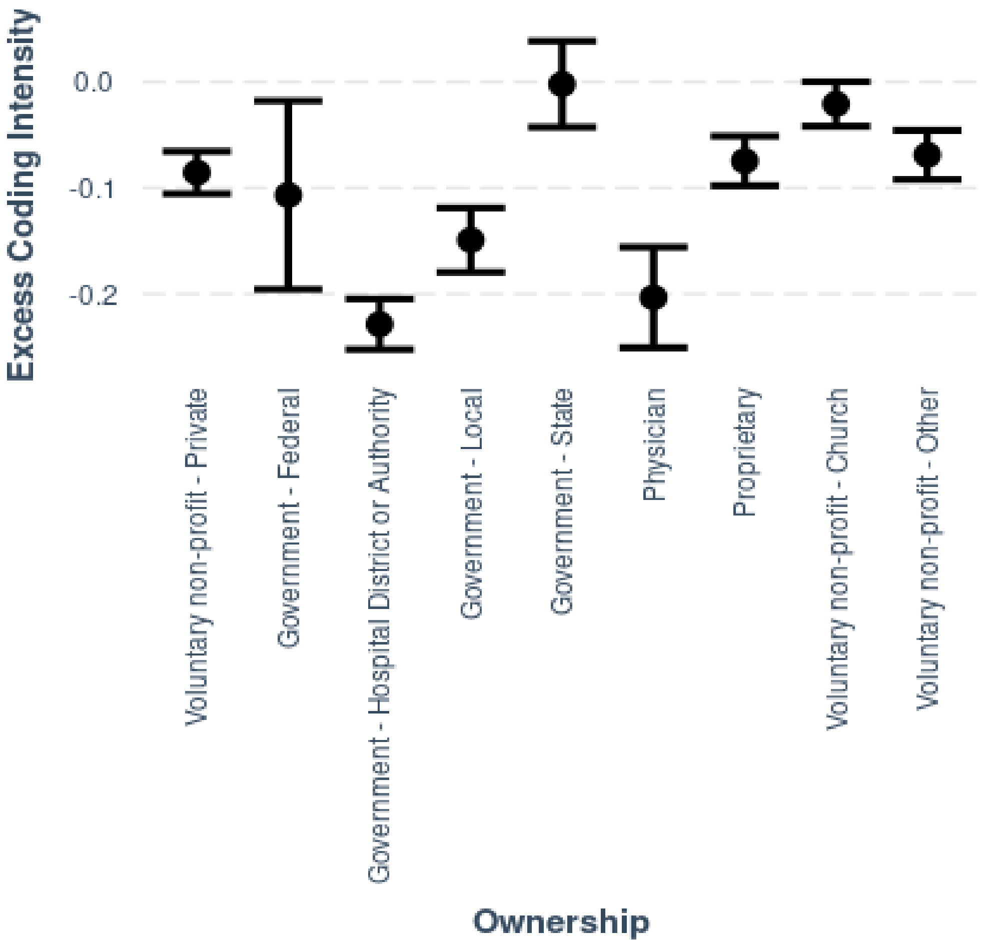

Figure S7. Effect plot of excess coding intensity by facility case mix index (CMI)

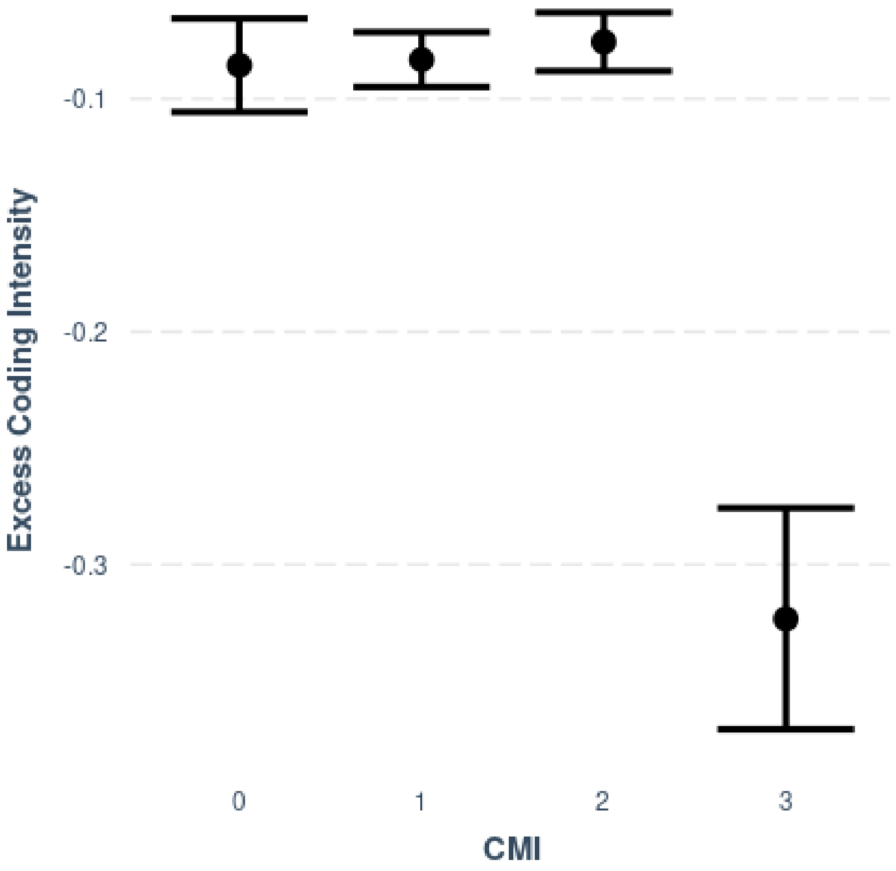

Figure S8. Effect plot of excess coding intensity by facility teaching status

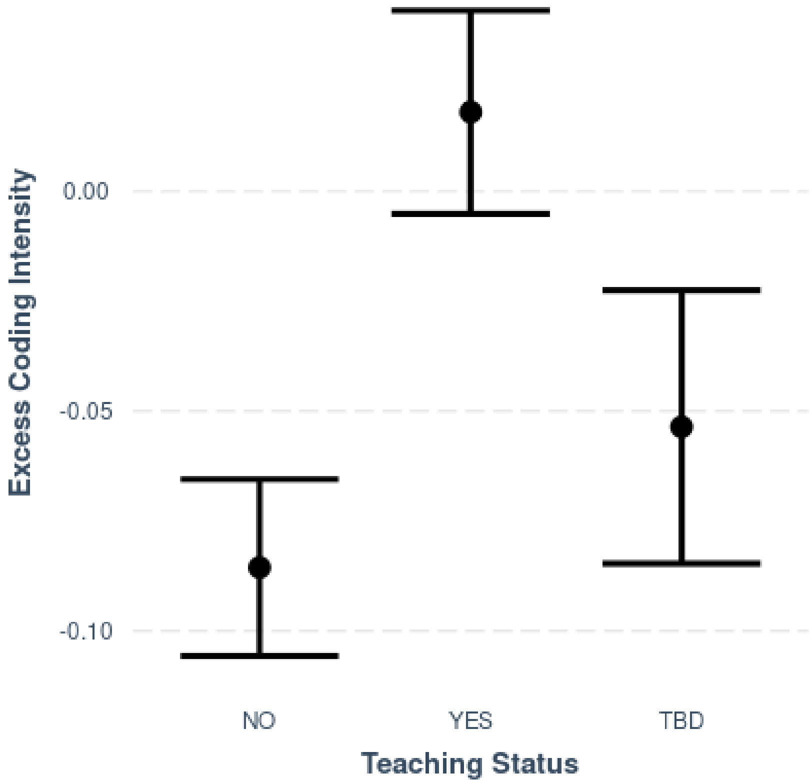

Figure S9. Effect plot of excess coding intensity by facility academic status

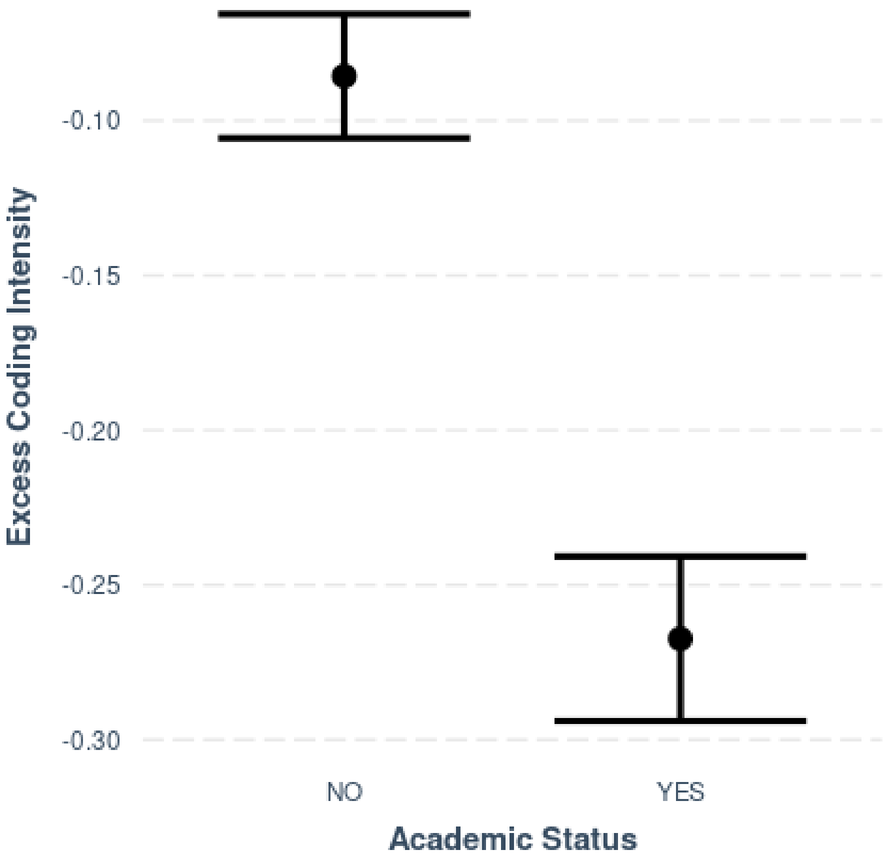

Figure S10. Effect plot of excess coding intensity by facility U.S. Census regional division

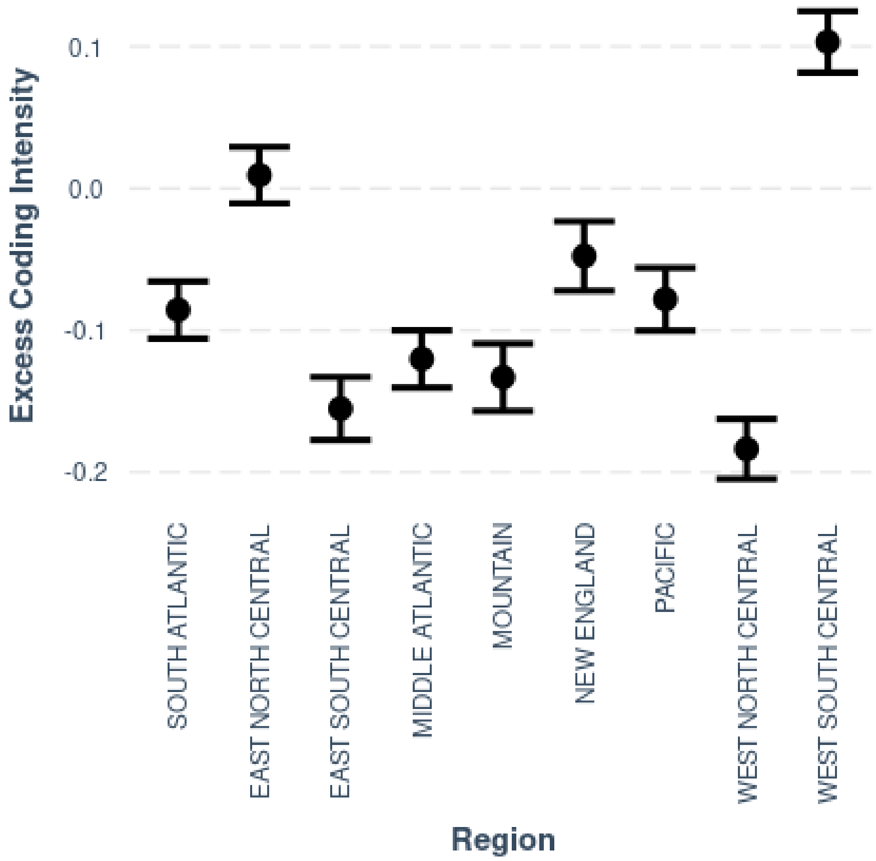

Figure S11. Effect plot of excess coding intensity by facility bed count

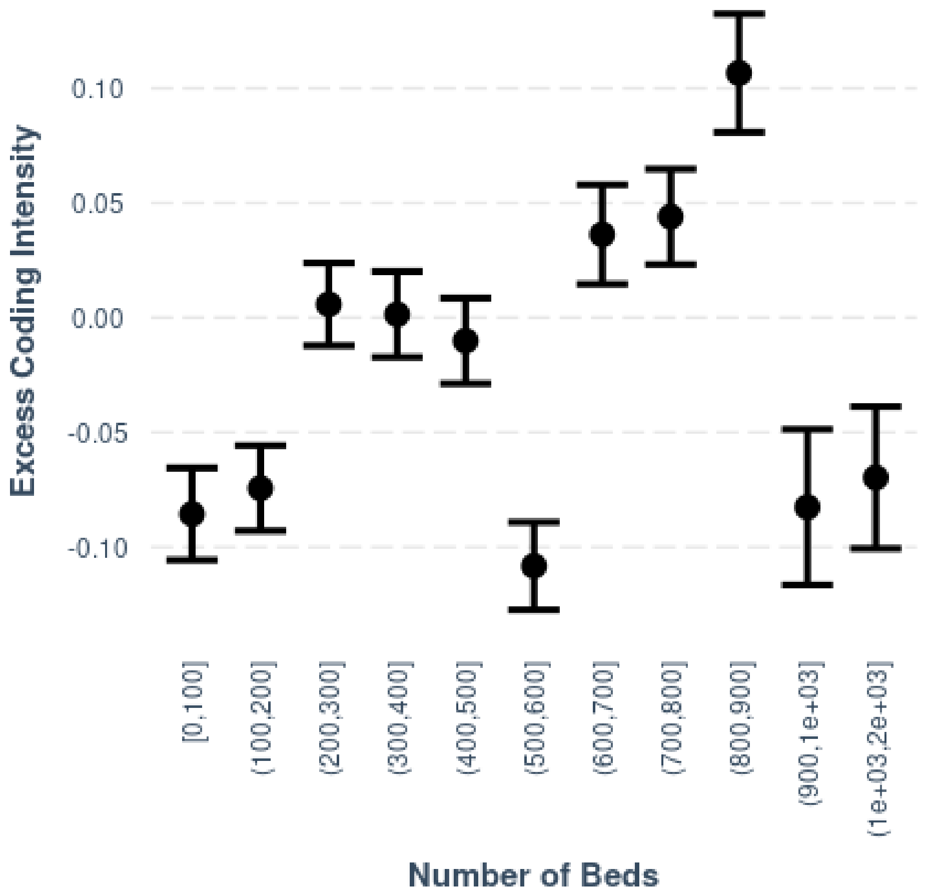

Figure S12. Heatmap of AAECI seasonality by admission month and facility region based on the U.S. Census Bureau definition of 9 regional divisions

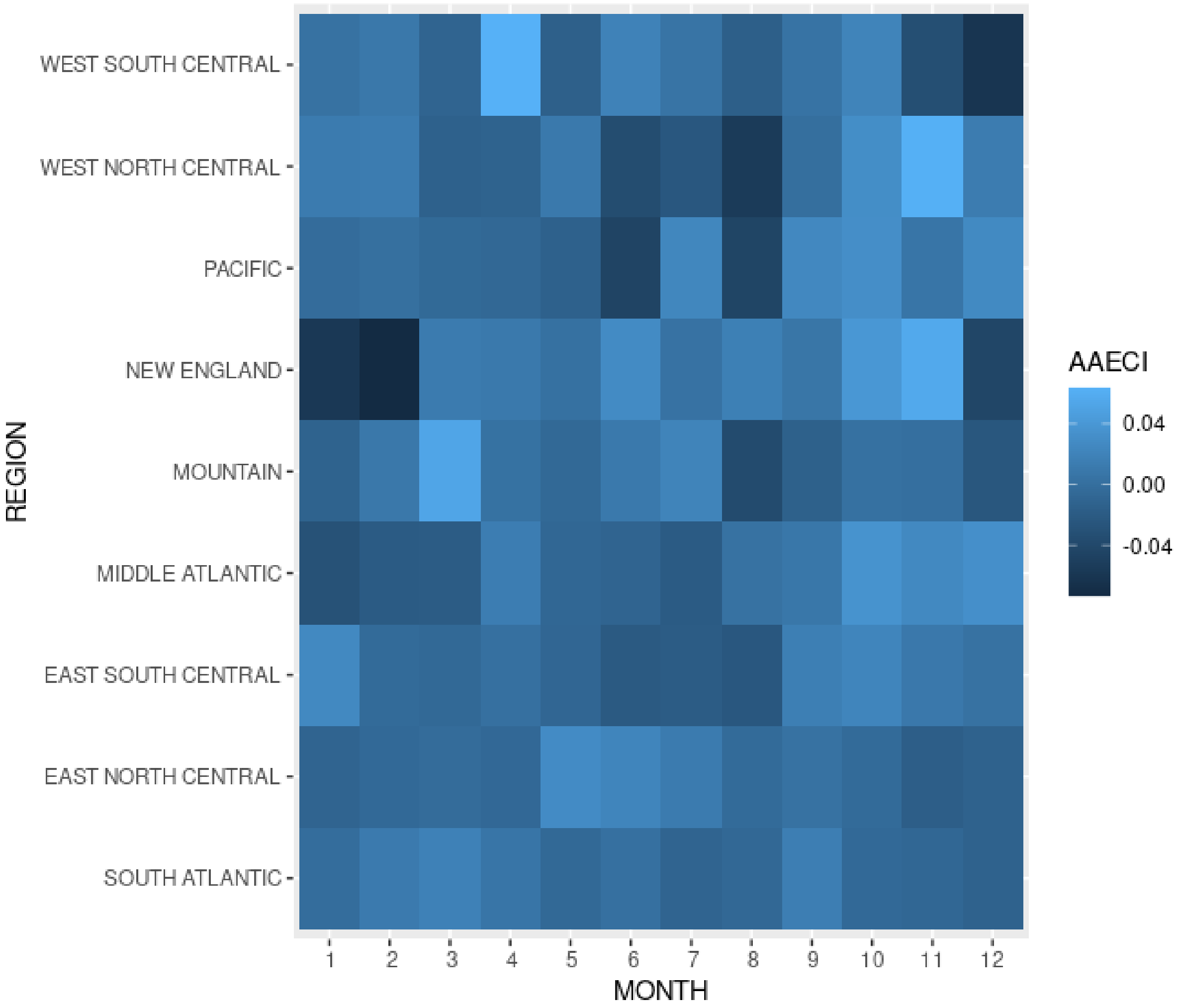

Figure S13. Effect plot of adjusted excess coding intensity (AECI) by facility

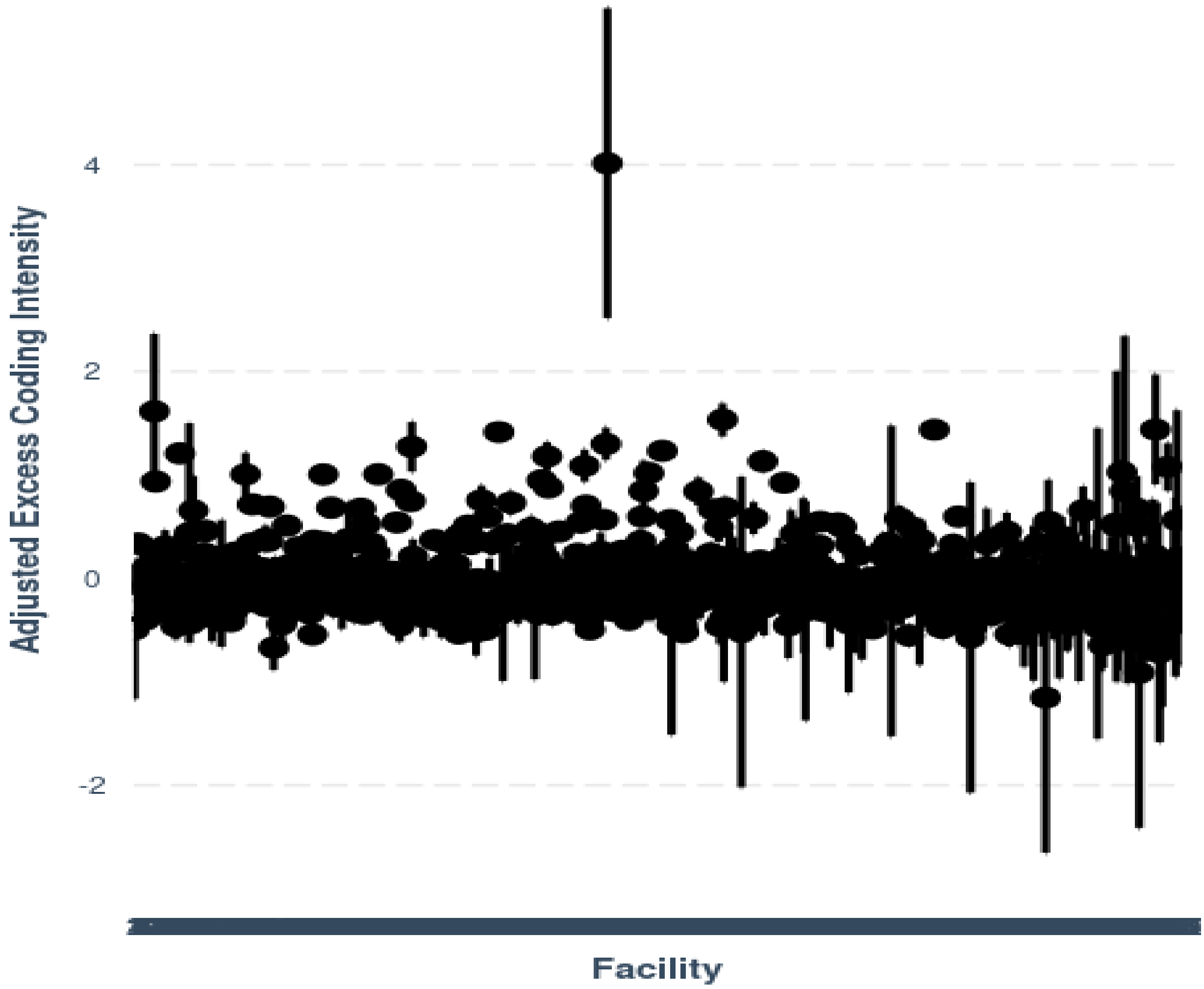

Figure S14. Sorted dot plot of adjusted excess coding intensity (AECI) across facilities

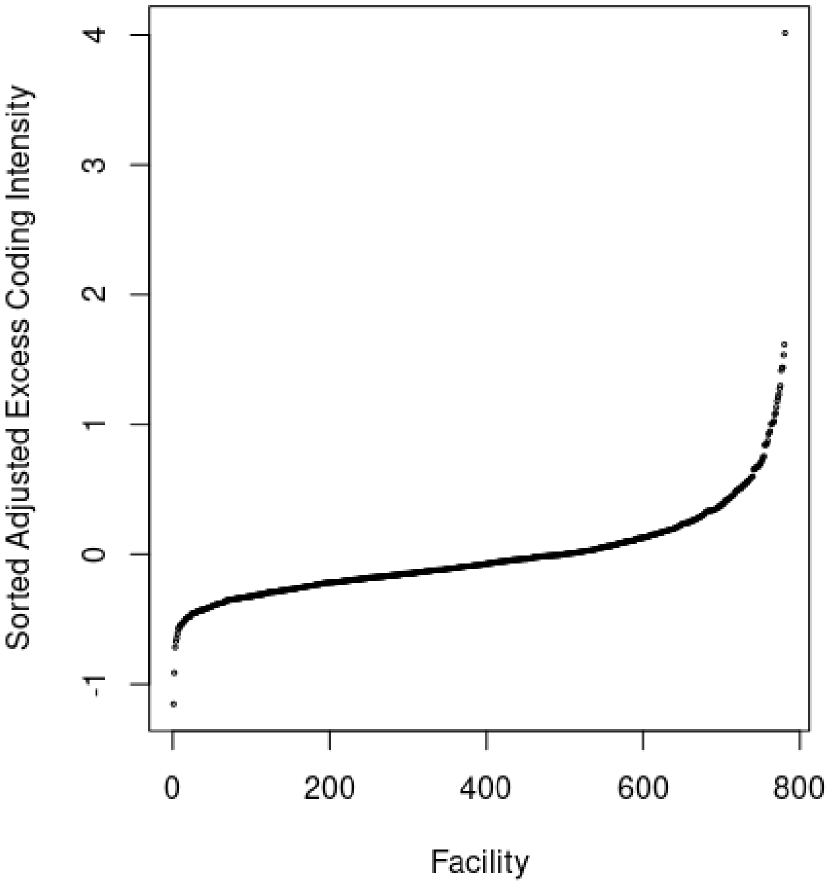

Supplement: Supplementary file 1 [file healthcare-10-01368-s001.zip › healthcare-1761987-supplementary.pdf]
